# Transcriptome Analysis Revealed the Molecular Response Mechanism of Non-heading Chinese Cabbage to Iron Deficiency Stress

**DOI:** 10.3389/fpls.2022.848424

**Published:** 2022-03-11

**Authors:** Jingping Yuan, Daohan Li, Changwei Shen, Chunhui Wu, Nadeem Khan, Feifei Pan, Helian Yang, Xin Li, Weili Guo, Bihua Chen, Xinzheng Li

**Affiliations:** ^1^School of Horticulture and Landscape Architecture, Henan Institute of Science and Technology, Xinxiang, China; ^2^Henan Engineering Research Center of the Development and Utilization of Characteristic Horticultural Plants, Xinxiang, China; ^3^School of Resource and Environmental Sciences, Henan Institute of Science and Technology, Xinxiang, China; ^4^Ottawa Research and Development Centre, Agriculture and Agri-Food Canada, Ottawa, ON, Canada; ^5^Department of Biology, University of Ottawa, Ottawa, ON, Canada

**Keywords:** non-heading Chinese cabbage, iron deficiency, transcriptome sequencing, differentially expressed genes, WGCNA

## Abstract

Iron is a trace metal that is found in animals, plants, and the human body. Human iron absorption is hampered by plant iron shortage, which leads to anemia. Leafy vegetables are one of the most direct and efficient sources of iron for humans. Despite the fact that ferrotrophic disorder is common in calcareous soil, however, non-heading Chinese cabbage performs a series of reactions in response to iron deficiency stress that help to preserve iron homeostasis *in vivo*. In this study, we discovered that iron deficiency stress caused leaf yellowing and impeded plant development in both iron-deficient and control treatments by viewing or measuring phenotypic, chlorophyll content, and Fe^2+^ content in both iron-deficient and control treatments. We found a total of 9213 differentially expressed genes (DEGs) in non-heading Chinese cabbage by comparing root and leaf transcriptome data with iron deficiency and control treatments. For instance, 1927 DEGs co-expressed in root and leaf, including 897 up-regulated and 1030 down-regulated genes, respectively. We selected some key antioxidant genes, hormone signal transduction, iron absorption and transport, chlorophyll metabolism, and transcription factors involved in the regulation of iron deficiency stress utilizing GO enrichment, KEGG enrichment, multiple types of functional annotation, and Weighted Gene Co-expression Network Analysis (WGCNA). This study identifies prospective genes for maintaining iron homeostasis under iron-deficient stress, offering a theoretical foundation for further research into the molecular mechanisms of greater adaptation to iron-deficient stress, and perhaps guiding the development of iron-tolerant varieties.

## Introduction

Iron is a trace metal that plays a role in a variety of physiological and metabolic processes, making it one of the most important trace elements for human health. As the major component of hemoglobin, iron’s most significant physiological role is to assist in oxygen intake, transport, and release (Zong et al., 2021). Anemia and other disorders have been linked to iron deficiency in studies (McLean et al., 2009). Furthermore, iron is a necessary trace element in plants and plays a vital role in their vegetative growth (Zhang et al., 2021). Iron, a component of hemoglobin and iron sulfur proteins such as cytochrome, catalase, peroxidase, and ferredoxin, has been discovered to have the potential to change the redox state. Photosynthesis, respiration, chlorophyll biosynthesis, nitrogen sulfur assimilation, and hormone biosynthesis are just a few of the fundamental cellular activities where iron plays a significant role (Briat et al., 2015; Connorton et al., 2017). Although iron is abundant in soils, it is rapidly oxidized to Fe^3+^, which is less soluble, reducing iron availability to plants (Li and Lan, 2017). Plants have evolved extensive adaptive mechanisms to maintain intracellular iron homeostasis in response to Fe^2+^ depletion through physiological, metabolic, and gene regulation over lengthy periods in response to Fe^2+^ deficit (Li and Lan, 2017).

Marschner et al. (1986) developed an approach I for Gemini plants and non-grass monocots to describe the adaptation process of plants in an iron-deficient environment. Plants release H^+^ to the rhizosphere via H^+^-ATPase (HA) on the plasma membrane during strategy I iron uptake to acidify the rhizosphere soil and enhance the solubility of Fe^3+^ in the soil. Plants use feroxoreductase (FRO) to convert Fe^3+^ to Fe^2+^ before allowing Fe^2+^ to enter the cell via the iron transporters HA, FRO, and IRT on the cell membrane (Marschner et al., 1986). The ergolic acid plant iron carrier MAs are the main ingredient for iron uptake by plants in Strategy II. Under iron deficiency stress, such plants release MAs, which have a significant affinity for Fe^3+^ and can form stable Fe^3+^-MAs trivalent chelates, which are then transported into plants (Marschner et al., 1986). Plants metal ion homeostasis maintenance still requires a complex signal transduction regulation mechanism. For instance involving many non-specific metal ion transport mechanisms, by distributing the excess metal ions, including iron, cell walls and other bubbles are not easy to cause damage area, partition these excess metal ions play a storage role at the same time (Zhang, 2012). Furthermore, plants are regulated by multiple phytohormones and signaling substances under iron deficiency stress (Matsuoka et al., 2014; Lucena et al., 2015; Kobayashi et al., 2016; Li et al., 2021).

Under iron deficiency stress, *AHA2*, *FRO2*, and *IRT1* have been discovered in *Arabidopsis thaliana*, a typical dicotyledonous plant (Zargar et al., 2015). Similarly, Pea (Cohen et al., 2004), cucumber (Waters et al., 2007), tomato (Zuchi et al., 2009), apple (Gao et al., 2011), and grape all have two important genes, *FRO2* and *IRT1* (Vannozzi et al., 2017).

In addition, plant responses to iron deficiency have been studied at the transcriptome, proteome, and metabolome levels (Forner-Giner et al., 2010; Yang et al., 2010). Key candidate genes ideal for iron deficiency stress, such as transcription regulators involved in iron stress regulation, have been identified rapidly using the above approaches, according to studies (Li et al., 2016; Connorton et al., 2017). Furthermore, during iron deficiency stress, plants are regulated by several plant hormones and signaling substances, primarily jasmonic acid (JA) (Kobayashi et al., 2016), gibberellins (Matsuoka et al., 2014), ethylene (Lucena et al., 2015), and reactive oxygen species (ROS) (Li et al., 2021).

To enhance human nutrition, finding a more cost-efficient, effective, and safe technique to supplement iron is necessary. Chinese cabbage is an essential non-staple food because it is a leafy vegetable crop. It is a key source of rich and inexpensive mineral nutrients for humans, such as iron and zinc (Wu et al., 2007). Chinese cabbage (*Brassica campestris* ssp. *chinensis*), also known as pakchoi, is a non-heading cabbage that originated in China. It is widely introduced and farmed in the north, accounting for 30 to 40% of the multiple cropping area of large and medium cities in the middle and lower parts of the Yangtze River (Tang et al., 2018). Therefore, increasing iron content in non-heading Chinese cabbage is of great significance to improve iron nutritional status in the human body.

Since most of the soil in northern China is calcareous, it is difficult for plants to absorb the iron they require, resulting in a significant drop in production and quality of non-heading Chinese cabbage (Rodriguez-Ramiro et al., 2019). Although some signal transduction effects of some key iron absorption and transport genes have been reported in model plants and other plants under iron deficiency stress (Marschner et al., 1986; Colangelo and Guerinot, 2004; Kobayashi et al., 2016; Hindt et al., 2017). However, the molecular regulatory mechanism of iron deficiency response in non-heading Chinese cabbage is largely unknown. As a result, the physiological changes of non-heading Chinese cabbage ‘Suzhouqing’ roots and leaves under iron deficiency were investigated. In addition, weighted gene co-expression network analysis (WGCNA) is very effective in interpreting the mechanism of abiotic stress (Panahi et al., 2019; Panahi and Hejazi, 2021). Transcriptome sequencing and WGCNA were also employed to uncover the likely response route of non-heading Chinese cabbage under iron deficiency stress. This study provides a theoretical foundation for further unraveling the molecular mechanism of important genes in non-heading Chinese cabbage iron homeostasis regulation, as well as some guidance for future non-heading Chinese cabbage varieties with high iron content.

## Materials and Methods

### Material Handling and Sample Collection of Non-heading Chinese Cabbage

The material employed was the non-heading Chinese cabbage ‘Suzhouqing,’ which was sown in a seedling tray containing a matrix-vermiculite (3:1) mixture and cultivated in a light incubator. The culture conditions were established at 24°C, 16 h of light/16°C, 8 h of darkness, and 65% relative humidity. The non-heading Chinese cabbage seedlings are moved to a turnover box with Hoagland nutrient solution for continuing culture during the four-leaf phase, and the nutrient solution is refreshed every 3 days. The experiment included a normal iron supply (control treatment) (CK, 0.02 mol/L) and an iron deficiency treatment (−Fe, 0 mol/L). On the 4th, 7th, 11th, and 14 days of culture in the turnover box, the phenotypes were observed. On the 14th day of cultivation, each treatment’s roots and leaves were collected simultaneously. The leaves were separated into three sections once they were harvested. The remaining samples were utilized to generate a transcriptome sequencing library, with one part used to determine chlorophyll content and one part used to determine Fe concentration. The remaining roots were similar to leaves and were used to generate the transcriptome sequencing library, with a portion of the root was utilized to determine the concentration of Fe.

### Method for Determination of Chlorophyll a, Chlorophyll b and Total Chlorophyll in Leaves of Non-heading Chinese Cabbage

We improved the prior Du et al. (2007) method for determining chlorophyll content in non-heading Chinese cabbage leaves. This is how it was done:

(1) Clean the surface dirt off of new non-heading Chinese cabbage leaves, remove the major veins, and cut the tissues; (2) Weigh three fresh cut samples of 0.1 g each for each treatment and deposit them in test tubes, respectively. To completely submerge the sample in an ethanol solution, 5 mL of 95% ethanol was added to each test tube. Soak it for 24 h in the dark; (3) The chlorophyll pigment extract was applied to the enzyme standard plate after 24 h in the dark, and 95% ethanol was employed as the control. The absorbance was measured at 665 nm and 649 nm; (4) Chlorophyll a and b concentrations (mg/L) were calculated using the formula, and the total chlorophyll (Chla + b) concentration was computed by adding the total sum of chlorophyll a (Chla) and chlorophyll b (Chlb).

The following formula was used for the chlorophyll quantification:

C_a_ = 13.95 A_665_-6.88 A_649_

C_b_ = 24.96 A_649_-7.32 A_665_

The concentrations of chlorophyll a, chlorophyll b, and total chlorophyll are represented by the formula Ca, Cb, and Ca + b. In 95% ethanol, the maximum absorption wavelengths of chlorophyll a and chlorophyll b were 665 nm and 649 nm, respectively. The absorbance of chlorophyll a and chlorophyll b were denoted by the letters A665 and A649, respectively.

(5) After determining the pigment concentration, the content of each pigment per unit fresh weight in the tissue was determined as follows:

Chloroplast pigment content (mg/g) = (pigment concentration × extract volume)/sample fresh weight

### Determination Method of Fe Content in Leaves and Roots of Non-heading Chinese Cabbage

The materials were non-heading Chinese cabbage roots and leaves that had been cultured for 14 days. The samples were first destroyed at 105°C before being dried at 70°C. The samples were finally crushed using a stainless steel grinding machine. The mixed acid (HNO_3_:HClO_4_ = 3:1) was used to digest the milled drying samples (0.25–0.5 mm sieve) 0.02–0.2 g. Finally, the digested solution was diluted to 100 mL with water, and the Fe concentration was measured using an Inductively Coupled Plasma Emission Spectrometer (ICP-AES).

### RNA Extraction, Library Construction and Transcriptome Sequencing

As samples, the roots and leaves from the control and iron deficiency treatments were obtained. Each treatment was carried out three times for a total of 12 samples. RNA was extracted using the RNA simple total RNA extraction kit, and the purity, concentration, and integrity of the RNA were determined using NanoDrop, a Qubit 2.0, and an Agilent 2100, respectively. The final cDNA library was created by reverse-transcribed total RNA of roughly 10 g. The Illumina NovaSeq™ 6000 was used to sequence the library after it had been qualified, and the sequencing read length was 2*150bp (PE150). Transcriptome data was submitted to the Sequence Read Archive (SRA) of the National Center for Biotechnology Information (NCBI) (Registration number PRJNA745363^1^).

### Sequencing Data Analysis and Differentially Expressed Gene Evaluation

Preprocessing the original data, including the elimination of sequencing joints (added during database development) and low-quality sequencing data (caused by the error of the sequencing instrument itself), is necessary to produce accurate and reliable analytical results. The effective data (Valid Data) was compared to the Chinese cabbage genome^2^ and the gene position information specified in the genome annotation file (gtf and gff) was statistically assessed. By counting the original sequencing, effective sequencing, Q20, Q30, and GC content (Ewing et al., 1998) for a comprehensive evaluation, we produce a large number of high-quality data through the Illumina high-throughput sequencing platform called raw data based on Sequencing By Synthesis (SBS) technology. StringTie was used to assemble the aforementioned reads in this study (Pertea et al., 2015). The expression levels of all transcripts were estimated using StringTie and DESeq2 (Love et al., 2014). Gene expression levels were determined using the Fragments Per Kilobase of Transcript per Million Fragments Mapped (FPKM) method (Florea et al., 2013), and differential expression genes (DEGs) were screened using the Fold Change ≥2 and False Discovery Rate (FDR) < 0.01 criteria (Florea et al., 2013). The FDR is calculated by subtracting the difference manifestness *p*-value from the error detection rate (Benjamini and Hochberg, 1995).

### Functional Classification and KEGG Analysis of Differentially Expressed Genes

Genes that have similar functions are often found together. The R language package is used to cluster the genes in the Java language environment, and the genes are then submitted to NCBI for GO item analysis. Biological process, molecular function, and cell component are the three major components of the GO annotation system. Enrichment in two ways *p*-value and adjusted *q*-value were used to analyze KEGG pathways. The KEGG database was compared to the NCBI’s BLAST search engine (Kanehisa et al., 2008).

### Weighted Gene Co-expression Network Analysis

The co-expression network is built using functions from the WGCNA package in R to describe the correlation patterns among genes in different samples (Langfelder and Horvath, 2008). To construct the adjacent matrix, all gene expression data is normalized using the log_2_ (1 + FPKM) value, and the soft threshold = 8 is chosen using the scale-free topology standard. The adjacency matrices were then turned into topological overlap matrices (TOM). Module required a minimum of 30 genes and a merge cut height of 0.25. Intergene variability was used to classify genes into clusters (Langfelder and Horvath, 2008). The correlation between the gene co-expression module and physiological indices was used to generate the correlation analysis (Liu et al., 2021). The protein interaction network of specific genes was constructed in the STRING database and visualized using Cytoscape 3.6.0 software (Shannon et al., 2003). Venn plots and heatmaps of DEGs were plotted using TBtools (Chen et al., 2020).

### Gene Validation Using Quantitative Real-time PCR

Nine genes were selected at random from DEGs in RNA-seq data to test the accuracy and reliability of transcriptome data. Primer 5.0 software (Ren et al., 2004) was used to design specific primers (Supplementary Table 9). RNA was reverse transcribed to cDNA from 1 μg of RNA using the PrimerScript RT Reagent Kit (Takara, Dalian, China). For quantitative real-time PCR (qRT-PCR), the cDNA was diluted a certain number of times. The reaction system included 10 μL of SYBR Green (Vazyme, Nanjing, China), 0.4 μL of forward primer, 0.4 μL of reverse primer, 0.4 μL of Rox, 2 μL of cDNA template, and 6.8 μL of re-evaporation. In each sample, three technical repeat tests were performed on nine potential genes selected from the transcriptome data. For each treated sample, three biological replicates were selected. The ABI StepOnePlus real-time fluorescence quantitative PCR machine was used to perform the qRT-PCR reaction. The reaction conditions were 95°C for 10 s, 95°C for 5 s, and 60°C for 30 s, for a total of 40 cycles. The dissolution curve conditions were reported by Yuan et al. (2019). To normalize the data, the *GAPDH* (glyceraldehyde 3-phosphate dehydrogenase) gene of non-heading Chinese cabbage was employed as an internal reference gene. The 2^–(ΔΔCT)^ method (Livak and Schmittgen, 2001) was used to calibrate the expression level of the target gene.

## Results

### Iron Deficiency Symptoms

**FIGURE 1:**
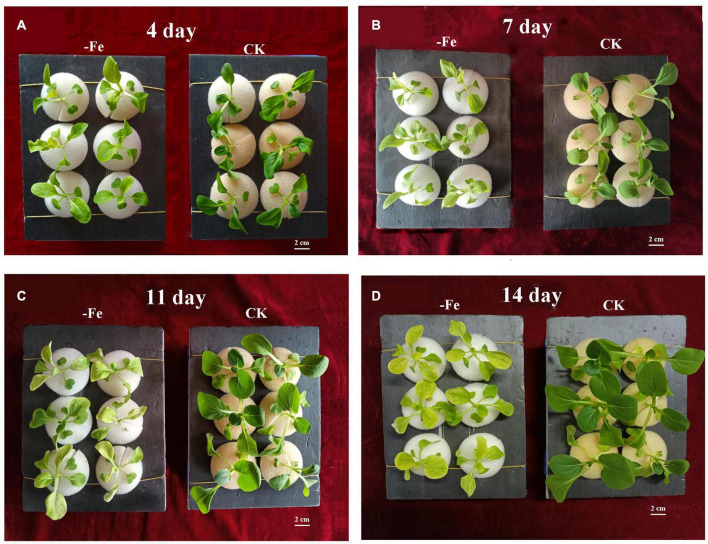
Non-heading Chinese cabbage seedlings’ responses to iron deficiency stress. **(A)** On the fourth day of iron deficit, phenotype of non-heading Chinese cabbage seedlings. **(B)** On the 7th day of iron deficit, phenotype of non-heading Chinese cabbage seedlings. **(C)** On the 11th day of iron deficit, phenotype of non-heading Chinese cabbage seedlings. **(D)** On the 14th day of iron deficit, phenotype of non-heading Chinese cabbage seedlings. Each treatment was repeated three times, and 6 plants were selected for each repetition. 2 cm high bar.

The non-heading Chinese cabbage ‘Suzhouqing’ was utilized as experimental material, with seedlings switched from matrix culture to nutrient solution culture at the four-leaf stage. The hydroponic experiment revealed that on the fourth day of hydroponics, there was no significant difference in plant height between the two treatments [iron deficiency treatment: 0 mol/L Fe^2+^; control (normal culture): 0.02 mol/L Fe^2+^], but the leaves under iron deficiency had a slight green loss compared to the control leaves (Figure 1A). The size and color of plant leave gradually changed as the amount of hydroponic time increased (Figures 1A–D). On the 14th day, the leaf area of non-heading Chinese cabbage was much larger than on the 4th and 7th days. We also discovered that at 14 days of culture, the leaf area of non-heading Chinese cabbage plants treated with iron deficiency was smaller than the control plants, and the leaf color was yellower than the control plants (Figure 1D). As a result, we hypothesized that iron deficiency promotes non-heading Chinese cabbage plant dwarfing and leaf yellowing.

### Physiological Indexes of Plants Under Iron Deficiency Stress

**FIGURE 2:**
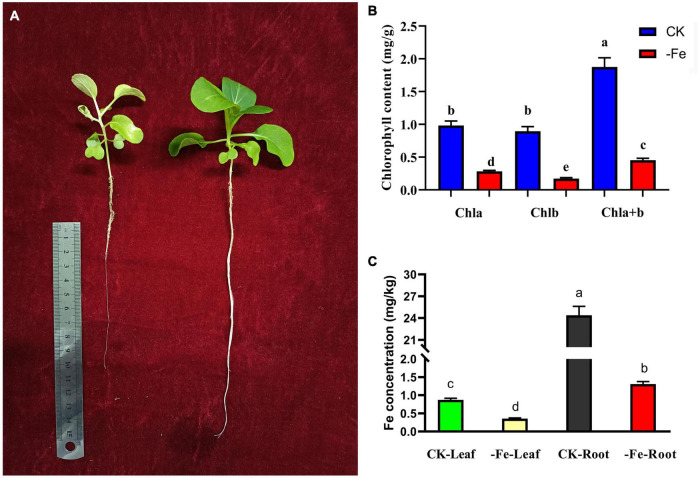
Analysis of chlorophyll content and iron content of non-heading Chinese cabbage under iron deficiency for 14 days. **(A)** Plant phenotype of non-heading Chinese cabbage seedlings after iron deficiency for 14 days. **(B)** Chlorophyll content in leaves of non-heading Chinese cabbage under iron deficiency and normal treatment. Chla, chlorophyll a; chlb, chlorophyll b; chla + b, the total content of chlorophyll a and chlorophyll b; For each treatment, 10 plants were selected for chlorophyll content assay. **(C)** Iron content in roots and leaves of non-heading Chinese cabbage under iron deficiency and normal treatment. For each sample, 3 plants were selected for iron content measurement.

The contents of chlorophyll and Fe^2+^ were examined to see how iron deficiency stress affected physiological indicators in non-heading Chinese cabbage. The materials utilized in this investigation were non-heading Chinese cabbage seedlings treated with iron deficiency and control for 14 days (Figure 2A). Under iron deficiency treatment, the contents of chlorophyll a, chlorophyll b, and chlorophyll a + b in seedling leaves dropped by 71.36, 80.83, and 75.88%, respectively (Figure 2B). Under iron deficiency treatment, the iron content of non-heading Chinese cabbage leaves and roots was reduced by 59.33 and 94.62%, respectively (Figure 2C). By analyzing the aboveground and underground parts of non-heading Chinese cabbage under two treatments, we discovered that iron deficiency not only inhibited the chlorophyll content and iron content in the aboveground tissue of non-heading Chinese cabbage, but also inhibited root growth and absorption of iron content (Figure 2).

### RNA-Seq Library Analysis

We conducted the transcriptome analysis of 12 samples (roots and leaves of control and iron deficiency treatment, each treatment repeated three times) using Illumina paired-end sequencing, yielding a total of 80.29 GB of clean data. Each sample yielded 6.31 GB of data, with a Q30 base percentage of 93.82% or higher (Supplementary Table 1). The alignment efficiency of each sample’s Clean Reads with the defined reference genome ranged from 66.56 to 87.53% (Supplementary Table 2). Twelve samples were given the name ‘treatment-tissue-number’ to make the analysis easier. For example, CK-Leaf-1 is the first sample in the leaves under normal conditions, whereas Fe-Root-2 is the second sample in the root under iron deficiency conditions (Supplementary Table 1).

### Overall Analysis of All Genes

**FIGURE 3:**
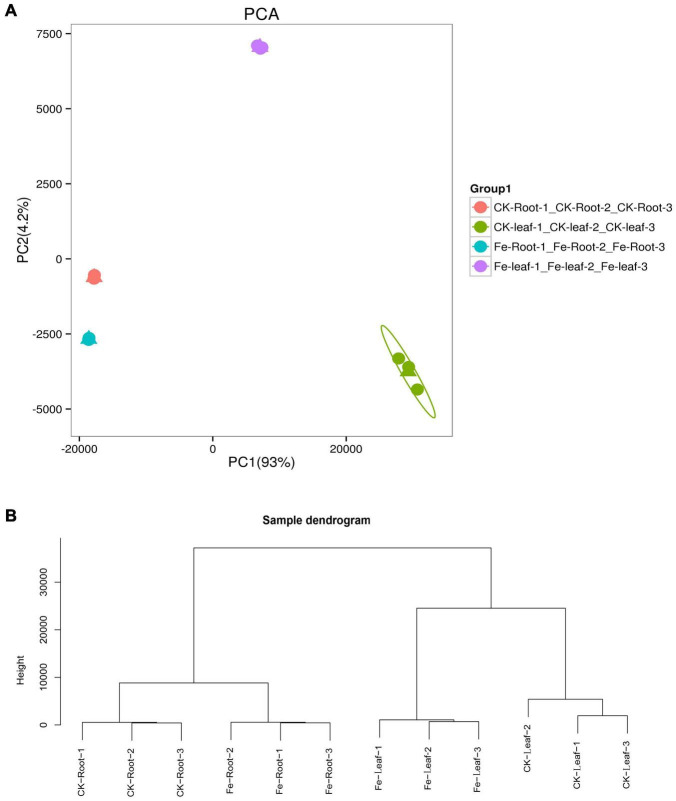
Principal component analysis and sample cluster analysis of all genes. **(A)** Principal component analysis of all genes in 12 libraries. **(B)** Cluster analysis of all samples in 12 libraries.

We discovered that principal component 1 (PCA1) described 93.0% of the content and principal component 2 (PCA2) explained 4.2% of the content of all genes in 12 libraries (Figure 3A). Each treatment’s three biological replicates were essentially clustered together, while CK Leaf, Fe Leaf, CK Root, and Fe Root were totally separated (Figure 3A). The results of the cluster analysis were similar to those of the main component analysis, indicating that the biological replication of the sample is consistent (Figure 3B).

**FIGURE 4:**
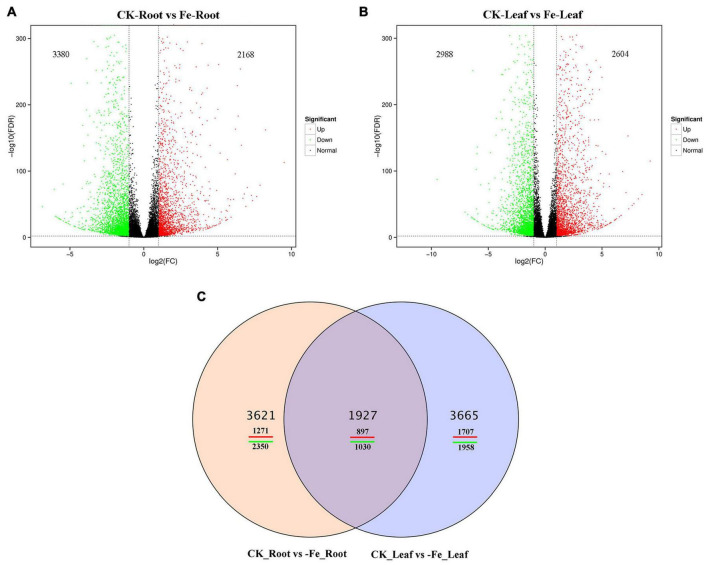
Differentially expressed genes in roots and leaves of non-heading Chinese cabbage responding to iron deficiency stress. **(A)** Volcanic map of differentially expressed genes in roots of non-heading Chinese cabbage under iron deficiency stress. **(B)** Volcanic map of differentially expressed genes in leaves of non-heading Chinese cabbage under iron deficiency stress. **(C)** Venn diagram of differentially expressed genes in roots and leaves under iron deficiency stress.

Transcriptome sequencing was performed on the samples of the two treatments to acquire DEGs in the leaves and roots of non-heading Chinese cabbage under iron deficiency stress and control treatment. We discovered 9213 DEGs in this investigation (Figure 4). In the roots of non-heading Chinese cabbage, 2168 genes were up-regulated and 3380 genes were down-regulated under iron deficiency stress (Figure 4A), whereas 2604 genes were up-regulated and 2988 genes were down-regulated in the leaves (Figure 4B). Under iron stress, a Venn diagram revealed that 1927 genes, including 897 up-regulated genes and 1030 down-regulated genes, were co-expressed in roots and leaves, revealing a link between DEGs in roots and leaves (Figure 4C). These findings imply that under iron deficiency stress, the root and leaf of non-heading Chinese cabbage may have a common or unique response mechanism.

### GO Classification and KEGG Enrichment of Differentially Expressed Genes

**FIGURE 5:**
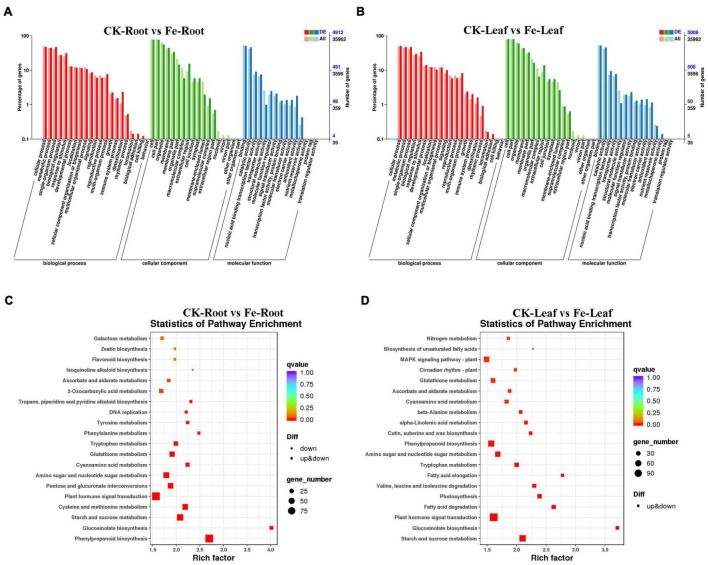
GO classification and KEGG enrichment analysis of DEGs in roots and leaves of non-heading Chinese cabbage under iron deficiency stress. **(A)** GO classification of DEGs in non-heading Chinese cabbage roots. **(B)** GO classification of DEGs in non-heading Chinese cabbage leaves, horizontal coordinate GO classification, ordinate left for the percentage of the number of genes, right for the number of genes. **(C)** KEGG enrichment analysis of DEGs in non-heading Chinese cabbage roots. **(D)** KEGG enrichment analysis of DEGs in non-heading Chinese cabbage leaves. Each block in the figure represents a KEGG pathway, the ordinate represents the name of the pathway, and the abscissa is the enrichment factor, indicating the ratio of the gene proportion annotated to a pathway in the differential gene to that annotated to the pathway in all genes. The greater the enrichment factor, the more obvious the enrichment level of differentially expressed genes in the pathway. The color of the block represents *q*-value, and *q*-value is the *p*-value corrected by multiple hypothesis tests. The smaller the *q*-value is, the more reliable the enrichment of differentially expressed genes in this pathway is. The size of the circle indicates the number of genes enriched in the pathway. The larger the circle is, the more genes are.

GO categorization analysis of DEGs in roots and leaves was performed to define the key functional categories of DEGs. The majority of DEGs were found to be involved in cell processes, metabolic processes, cell/cell components, binding sites, and catalytic activities, according to the findings (Figures 5A,B and Supplementary Table 3).

Differentially expressed genes were also examined for KEGG enrichment in this research (Supplementary Table 4). DEGs were found to be confined in plant hormone signal transduction (Ko04075), phenylpropane-like biosynthesis (Ko00940), starch and sucrose metabolism (Ko00500), plant-pathogen interaction (Ko04626), cysteine and methionine metabolism (Ko00270), and other pathways in the roots of non-heading Chinese cabbage under iron deficiency stress (Figure 5C). In the leaves of non-heading Chinese cabbage under iron deficiency stress, DEGs are mainly concentrated in plant hormone signal transduction (Ko04075), carbon metabolism (Ko01200), starch and sucrose metabolism (Ko00500), phenylpropanoid biosynthesis (Ko00940), amino acid biosynthesis (Ko01230) and other pathways (Figure 5D).

### Reactive Oxygen Species Analysis Under Iron Deficiency Stress

Many DEGs are linked to antioxidant enzyme activity, such as peroxidase (POD), superoxide dismutase (SOD), catalase (CAT), and ascorbate peroxidase (APX), were discovered during iron deficiency stress (Table 1). Under iron deprivation, the transcript profiles of 58 *POD* genes altered considerably in both leaves and roots, with 7 genes up-regulated and 32 genes down-regulated. Only in leaves were 5 *POD* genes highly up-regulated and 5 genes significantly down-regulated. In both roots and leaves, four *POD* genes were considerably up-regulated and five genes were significantly down-regulated (Supplementary Figure 1A). Three *SOD* genes were found to be considerably up-regulated exclusively in leaves, whereas one gene (*Bra013863*) was found to be strongly down-regulated in both leaves and roots (Supplementary Figure 1B). In addition, two *CAT* genes (*Bra034674* and *Bra034674*) were highly up-regulated in leaves, while one *CAT* gene (*Bra034674*) was strongly down-regulated. In both leaves and roots, one *CAT* gene (*Bra025832*) was considerably down-regulated (see Supplementary Figure 1C). Only one *APX* gene (*Brassica rapa*_ *newGene_2429*) was down-regulated significantly in roots, and one *APX* gene (*Bra031598*) was down-regulated strongly in both roots and leaves (Supplementary Figure 1D).

**TABLE 1:** Key antioxidant enzyme genes of non-heading Chinese cabbage under iron deficiency stress.

Gene_ID	Gene name	Module	CK-Root vs. Fe-Root DESeq2_FDR	CK-Root vs. Fe-Root DESeq2_log_2_FC	CK-Root vs. Fe-Root DESeq2_(FDR_0.01_FC_2)_regulated	CK-leaf vs. Fe-leaf DESeq2_FDR	CK-leaf vs. Fe-leaf DESeq2_log_2_FC	CK-leaf vs. Fe-leaf DESeq2_(FDR_0.01_FC_2)_regulated	NR_annotation
Bra002271.gene	POD N	blue	0.00	1.35	up	−	−	−	PREDICTED: peroxidase N [Brassica rapa]
Bra001084.gene	POD 28	blue	2.04E-142	1.12	up	−	−	−	PREDICTED: peroxidase 28-like [Brassica rapa]
Bra002270.gene	POD 58	blue	2.23E-41	1.91	up	−	−	−	PREDICTED: peroxidase 58 [Brassica rapa]
Bra009105.gene	POD 52	blue	3.39E-16	1.01	up	2.44E-02	0.74	normal	peroxidase P7 isoform X1 [Brassica napus]
Bra009221.gene	POD 54	blue	1.06E-123	1.32	up	1.77E-100	2.99	up	PREDICTED: peroxidase A2 [Brassica rapa]
Bra011438.gene	POD 47	blue	6.05E-59	0.75	normal	6.55E-05	1.32	up	PREDICTED: peroxidase 47 [Brassica rapa]
Bra014200.gene	POD 10	blue	0.00	1.64	up	−	−	−	PREDICTED: peroxidase 10 [Brassica rapa]
Bra017120.gene	POD C3	blue	0.00	1.87	up	0.00	4.88	up	PREDICTED: peroxidase C3-like [Brassica rapa]
Bra024268.gene	POD 70	blue	4.76E-47	2.92	up	−	−	−	PREDICTED: peroxidase 70 [Brassica rapa]
Bra037007.gene	POD 47	blue	0.00	2.50	up	0.00	0.53	normal	PREDICTED: peroxidase 47 [Brassica rapa]
Bra037877.gene	POD C2	blue	0.00	1.65	up	0.00	1.99	up	PREDICTED: peroxidase C2 [Brassica rapa]
Bra015403.gene	POD 2	brown	−	−	−	1.29E-118	−3.07	down	PREDICTED: peroxidase 2-like [Brassica rapa]
Bra017830.gene	POD 50	brown	7.50E-01	−0.14	normal	3.49E-37	−1.29	down	peroxidase 50 isoform X1 [Brassica napus]
Bra034561.gene	POD 47	brown	−	−	−	8.05E-132	−1.46	down	PREDICTED: peroxidase 47-like [Brassica rapa]
Bra011771.gene	POD 50	green	1.14E-02	−0.16	normal	9.54E-132	1.39	up	PREDICTED: peroxidase 50 [Brassica rapa]
Bra003918.gene	POD 12	green yellow	2.73E-03	−1.40	down	7.21E-01	−0.04	normal	PREDICTED: peroxidase 12-like [Brassica rapa]
Bra024269.gene	POD 71	red	2.98E-82	1.68	up	3.56E-124	3.39	up	PREDICTED: peroxidase 71 [Brassica rapa]
Bra030241.gene	POD 17	red	1.25E-05	0.88	normal	3.66E-07	1.06	up	PREDICTED: peroxidase 17 [Brassica rapa]
Bra033040.gene	POD 31	red	3.85E-05	−1.26	down	3.37E-08	−0.95	normal	PREDICTED: peroxidase 31 [Brassica rapa]
Bra000058.gene	POD 22	turquoise	0.00	−2.11	down	−	−	−	PREDICTED: peroxidase 22-like [Brassica rapa]
Bra000113.gene	POD 24	turquoise	3.53E-29	−5.74	Down	−	−	−	PREDICTED: peroxidase 24 [Brassica rapa]
Bra001986.gene	POD 64	turquoise	1.19E-79	−2.09	down	−	−	−	PREDICTED: peroxidase 64 [Brassica rapa]
Bra004343.gene	POD 11	turquoise	1.32E-27	−1.52	down	−	−	−	PREDICTED: peroxidase 11 [Brassica rapa]
Bra005346.gene	POD 20	turquoise	3.42E-19	−1.52	down	−	−	−	PREDICTED: peroxidase 20 [Brassica rapa]
Bra008775.gene	POD 55	turquoise	1.04E-12	−1.76	down	−	−	−	peroxidase 55-like [Brassica napus]
Bra009728.gene	POD 61	turquoise	1.11E-23	−2.95	down	−	−	−	PREDICTED: probable peroxidase 61 [Brassica rapa]
Bra011170.gene	POD 45	turquoise	2.40E-172	−2.33	down	−	−	−	PREDICTED: peroxidase 45 [Brassica rapa]
Bra011681.gene	POD 49	turquoise	1.77E-160	−1.42	down	−	−	−	PREDICTED: peroxidase 49 [Brassica rapa]
Bra011683.gene	POD 49	turquoise	3.57E-211	−1.47	down	−	−	−	PREDICTED: peroxidase 49-like [Brassica rapa]
Bra013576.gene	POD 42	turquoise	1.63E-94	−0.48	normal	0.00	−1.87	down	PREDICTED: peroxidase 42 [Brassica rapa]
Bra013943.gene	POD 44	turquoise	2.32E-40	−2.98	down	−	−	−	PREDICTED: peroxidase 44 [Brassica rapa]
Bra015402.gene	POD 2	turquoise	4.09E-28	−5.68	down	−	−	−	PREDICTED: peroxidase 2-like [Brassica rapa]
Bra015404.gene	POD 3	turquoise	4.51E-75	−2.35	down	3.98E-21	−1.88	down	PREDICTED: peroxidase 3-like [Brassica rapa]
Bra016127.gene	POD 12	turquoise	5.11E-42	−0.60	normal	3.80E-121	−1.46	down	PREDICTED: peroxidase 12 [Brassica rapa]
Bra017761.gene	POD 49	turquoise	0.00	−2.38	down	−	−	−	PREDICTED: peroxidase 49 [Brassica rapa]
Bra019132.gene	POD 44	turquoise	2.94E-45	−4.26	down	−	−	−	PREDICTED: peroxidase 44-like [Brassica rapa]
Bra020370.gene	POD 67	turquoise	2.33E-15	−1.85	down	−	−	−	PREDICTED: peroxidase 67 [Brassica rapa]
Bra021488.gene	POD 27	turquoise	3.51E-22	−2.86	down	−	−	−	PREDICTED: peroxidase 27-like [Brassica rapa]
Bra022567.gene	POD 66	turquoise	1.60E-58	−2.38	down	4.68E-14	−1.60	down	PREDICTED: peroxidase 66 [Brassica rapa]
Bra023099.gene	POD 21	turquoise	1.52E-148	−1.35	down	2.97E-03	0.42	normal	PREDICTED: peroxidase 21 isoform X1 [Brassica rapa]
Bra023509.gene	POD 56	turquoise	9.14E-51	−1.27	down	−	−	−	PREDICTED: peroxidase 56-like [Brassica rapa]
Bra024033.gene	POD 48	turquoise	5.98E-26	−1.92	down	6.74E-04	−2.37	down	PREDICTED: putative Peroxidase 48 [Brassica rapa]
Bra024102.gene	POD 45	turquoise	0.00	−3.61	down	−	−	−	PREDICTED: peroxidase 45 [Brassica rapa]
Bra025604.gene	POD 63	turquoise	1.39E-19	−1.19	Down	1.43E-10	−1.62	down	PREDICTED: peroxidase 63 [Brassica rapa]
Bra028285.gene	POD 66	turquoise	2.94E-15	−1.81	down	3.16E-02	−0.83	normal	PREDICTED: peroxidase 66 [Brassica rapa]
Bra030606.gene	POD 2	turquoise	8.70E-38	−4.85	down	−	−	−	PREDICTED: peroxidase 2-like [Brassica rapa]
Bra030607.gene	POD 2	turquoise	1.51E-13	−2.18	down	−	−	−	PREDICTED: peroxidase 2-like [Brassica rapa]
Bra031309.gene	POD 30	turquoise	7.56E-266	−1.54	down	1.48E-01	0.33	normal	peroxidase 30 [Brassica napus]
Bra031934.gene	POD 69	turquoise	1.51E-71	−1.05	down	−	−	−	PREDICTED: peroxidase 69-like [Brassica rapa]
Bra032474.gene	POD 3	turquoise	3.85E-147	−1.32	down	−	−	−	PREDICTED: LOW QUALITY PROTEIN: peroxidase 3-like [Brassica rapa]
Bra032477.gene	POD 2	turquoise	1.05E-30	−4.93	down	−	−	−	PREDICTED: peroxidase 2-like [Brassica rapa]
Bra035235.gene	POD 39	turquoise	1.01E-196	−1.26	down	−	−	−	PREDICTED: peroxidase 39 [Brassica rapa]
Bra038345.gene	POD 11	turquoise	1.72E-15	−1.27	down	−	−	−	PREDICTED: peroxidase 11-like [Brassica rapa]
Bra039059.gene	POD 31	turquoise	6.88E-66	−1.42	down	1.61E-45	−1.47	down	PREDICTED: peroxidase 31-like [Brassica rapa]
Bra039137.gene	POD 27	turquoise	6.39E-188	−3.17	down	−	−	−	PREDICTED: peroxidase 27 [Brassica rapa]
Bra041169.gene	POD 49	turquoise	4.98E-04	−1.08	down	−	−	−	PREDICTED: peroxidase 49 [Brassica rapa]
Bra029933.gene	POD 34	yellow	1.66E-08	0.57	normal	0.00	2.45	up	PREDICTED: peroxidase 34 [Brassica rapa]
Bra031933.gene	POD 71	yellow	6.70E-02	0.33	normal	3.51E-25	1.22	up	PREDICTED: peroxidase 71-like [Brassica rapa]
Bra031642.gene	SODC	brown	6.04E-01	−0.03	normal	2.06E-73	1.14	up	PREDICTED: superoxide dismutase [Cu-Zn] [Brassica rapa]
Bra013863.gene	SOD	green	0.00	−2.32	down	0.00	−12.02	down	superoxide dismutase [Fe] 1, chloroplastic-like [Brassica napus]
Bra026968.gene	SODC	red	4.66E-24	0.91	normal	4.90E-21	1.02	up	PREDICTED: copper chaperone for superoxide dismutase, chloroplastic/cytosolic-like [Brassica rapa]
Bra034394.gene	SODC	yellow	5.39E-06	−0.22	normal	1.07E-132	1.08	up	copper/zinc superoxide dismutase, partial [Brassica rapa]
Bra025832.gene	CAT 3	brown	1.28E-31	−1.24	down	0.00	−3.63	down	PREDICTED: catalase-3 [Brassica rapa]
Bra034674.gene	CAT 2	brown	−	−	−	1.05E-214	−1.28	down	PREDICTED: catalase-2 isoform X2 [Brassica rapa]
Bra011584.gene	CAT 2	yellow	2.06E-03	−0.39	normal	4.50E-66	1.59	up	PREDICTED: catalase-2 [Brassica rapa]
Bra012239.gene	CAT 1	yellow	3.08E-08	0.46	normal	2.30E-279	1.82	up	PREDICTED: catalase-1 [Brassica rapa]
Bra031598.gene	APX	turquoise	1.99E-156	−1.49	down	1.29E-139	−2.01	down	PREDICTED: L-ascorbate peroxidase 1, cytosolic [Brassica rapa]
Brassica_rapa_newGene_2429	APX	turquoise	2.71E-46	−1.91	down	4.21E-01	−0.24	normal	L-ascorbate peroxidase 5, peroxisomal-like [Brassica napus]

*CK root vs. Fe root: the treatment of iron deficiency in roots was compared with the normal treatment; CK leaf vs. Fe leaf: the treatment of iron deficiency in leaf was compared with the normal treatment; DESeq2_ FDR, false discovery rate of differential gene expression; up, up regulation; down, down regulation; normal, has no change.*

### Iron Deficiency Stress Regulates the Plant Hormones

Iron deficiency in plant seedlings influence the signaling system for plant hormones. Under iron deficiency stress, 1 *SAUR* gene, 10 *AUX/IAA* genes, 3 *GH3* genes, and 4 *Auxin transporter* genes were considerably down-regulated in both roots and leaves, whereas 11 *SAUR* genes and 1 *AUX/IAA* gene were significantly down-regulated exclusively in leaves. Eight *GH3* genes were strongly down-regulated in roots under iron deficiency stress, but only one gene (*Bra022725*) was highly up-regulated in leaves, and three genes’ expression was dramatically down-regulated (Supplementary Table 5). Three *ARR* genes were considerably up-regulated and four of them were significantly down-regulated in the cytokinin signaling pathway in both roots and leaves. Similarly, only one *ARR* gene (*Bra002512*) was highly up-regulated in roots, whereas two others were significantly down-regulated. Also, three *ARR* genes were highly upregulated exclusively in leaves, while nine were considerably downregulated only in leaves (Supplementary Table 5). Under iron deficiency stress, the expression patterns of 10 *PYL* genes associated with the abscisic acid signal pathway changed considerably. One gene (*Bra005115*) was significantly down-regulated in both roots and leaves, and seven genes were exclusively significantly down-regulated in leaves. Under iron deficiency stress, only one *JAR* gene (*Bra034205*) associated with jasmonic acid signal was discovered to be significantly down-regulated in both roots and leaves (Supplementary Table 5). According to the findings, iron deficiency in Chinese cabbage seedlings had an impact on plant hormone signaling pathways, particularly auxin (IAA), cytokinin (CTK), abscisic acid (ABA), and jasmonic acid (JA).

### Candidate Gene Set of Iron Deficiency Stress Based on Weighted Gene Co-expression Network Analysis

**FIGURE 6:**
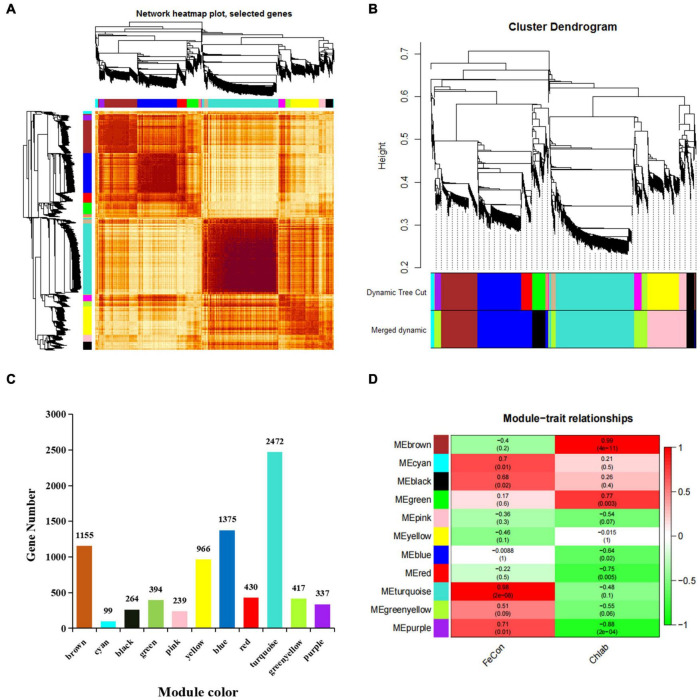
Gene co-expression modules of 8148 DEGs were analyzed based on WGCNA. **(A)** Gene expression clustering tree and co-expression topological heatmap. **(B)** Hierarchical clustering trees and modules for 8148 differentially expressed genes. The main tree branches are represented by different colors. There are 14 modules before optimization and 11 modules after optimization and fusion. Each leaf on a tree represents a gene. **(C)** The number of genes corresponding to the merged modules, a total of 11 modules, different colors represent different modules. **(D)** Correlation between different modules and iron content in leaves and roots and chlorophyll a + b content in leaves (correlation coefficient and *p*-value). FeCon represents iron content in leaves and roots; chlab indicates chlorophyll a + b content in leaves; different colors on the left represent different modules.

To better understand the co-expression network of associated genes in non-heading Chinese cabbage roots and leaves under iron deficiency stress, 9213 DEGs were screened by transcriptome sequencing, and 8148 DEGs were selected for WGCNA analysis (Supplementary Table 6). Gene expression cluster tree (Figure 6A) and hierarchical cluster tree analysis (Figure 6B) in WGCNA yielded a total of 11 modules. The largest module, with 2472 genes, accounted for 30.34% of all DEGs, whereas the smallest module, with 99 genes, accounted for 1.22% of all DEGs (Figure 6C). The physiological index of iron content (FeCon) was strongly positively correlated with the MEturquoise module, according to correlation analysis between these modules and iron content or chlorophyll a + b content (chlab). A total of 2319 genes were within the correlation coefficient >0.75, *p* < 0.01. Chlab was positively correlated with MEbrown and MEgreen modules, and negatively correlated with MEred and MEpurple modules. There were 968, 95, 118, and 18 genes in MEbrown, MEgreen, Mered, and MEpurple modules, respectively, with correlation coefficients >0.75, *p* < 0.01 (Figure 6D and Supplementary Table 6).

### Differentially Expressed Genes Related to Iron Absorption and Transport

To further screen genes involved in iron uptake and transport, 40 DEGs were obtained by searching the keyword ‘iron’ in 8148 DEGs (Table 2), including iron reductase protein (6 genes), bivalent iron transporter (6 genes), vacuolar iron transporter (5 genes), metal nicotinamide transporter (5 genes), ferritin (4 genes), proton ATPase (4 genes), iron deficiency-induced transcription factors (3 genes), zinc transporter (3 genes), metal transporter (3 genes), and oligopeptide transporter (1 gene) (Table 2). Among the 40 DEGs, we found that they came from many modules in WGCNA, of which 9 genes from MEturquoise module, were *FRO8* (*Bra023239*), *VIT2* (*Bra008277*, *Bra015718*), *VIT1* (*Bra012271*, *Bra025879*), *YSL2* (*Bra009756*), *HA* (*Bra027752*, *Bra040512*), and *ZIP2* (*Bra020314*). As a result, we assume that these nine genes are important in iron uptake and transport.

**TABLE 2:** Differentially expressed genes related to iron absorption and transport in non-heading Chinese cabbage under iron deficiency stress.

Gene_ID	Gene name	Module	CK-Root vs. Fe-Root DESeq2_FDR	CK-Root vs. Fe-Root DESeq2_log_2_FC	CK-Root vs. Fe-Root DESeq2_(FDR_0.01_FC_2)_regulated	CK-leaf vs. Fe-leaf DESeq2_FDR	CK-leaf vs. Fe-leaf DESeq2_log_2_FC	CK-leaf vs. Fe-leaf DESeq2_(FDR_0.01_FC_2)_regulated	Annotation
Bra023239.gene	FRO8	turquoise	1.26E-43	−1.21	down	4.68E-40	−1.37	down	PREDICTED: ferric reduction oxidase 8, mitochondrial isoform X2 [Brassica rapa]
Bra032640.gene	FRO2	blue	0.00	3.65	up	6.55E-03	−0.35	normal	PREDICTED: ferric reduction oxidase 2 [Brassica rapa]
Bra032641.gene	FRO1	cyan	8.50E-293	3.94	up	4.48E-43	1.55	up	PREDICTED: probable ferric reduction oxidase 1 isoform X3 [Brassica rapa]
Bra033252.gene	FRO2	blue	0.00	4.81	up	7.93E-02	0.14	normal	PREDICTED: ferric reduction oxidase 2 [Brassica rapa]
Bra037329.gene	FRO2	blue	0.00	4.64	up	−	−	−	PREDICTED: ferric reduction oxidase 2-like [Brassica rapa]
Bra037953.gene	FRO7	brown	4.02E-01	−0.36	normal	4.73E-213	−1.18	down	PREDICTED: ferric reduction oxidase 7, chloroplastic [Brassica rapa]
Bra012534.gene	IRT1	blue	5.06E-164	6.20	up	−	−	−	PREDICTED: fe(2 +) transport protein 1-like [Brassica rapa]
Bra012535.gene	IRT2	blue	1.58E-39	3.62	up	−	−	−	PREDICTED: fe(2 +) transport protein 2-like [Brassica rapa]
Bra013419.gene	IRT1	blue	0.00	4.85	up	−	−	−	PREDICTED: fe(2 +) transport protein 1-like [Brassica rapa]
Bra013420.gene	IRT1	blue	1.70E-218	3.99	up	−	−	−	PREDICTED: fe(2 +) transport protein1-like [Brassica rapa]
Bra013422.gene	IRT1	blue	1.38E-229	6.33	up	−	−	−	PREDICTED: fe(2 +) transport protein 1 [Brassica rapa]
Bra027108.gene	IRT3	green yellow	3.10E-01	0.17	normal	5.83E-05	−1.29	down	PREDICTED: fe(2 +) transport protein 3, chloroplastic [Brassica rapa]
Bra008277.gene	VIT2	turquoise	5.11E-11	−1.80	down	3.99E-02	−1.53	normal	PREDICTED: vacuolar iron transporter homolog 2 [Brassica rapa]
Bra012271.gene	VIT1	turquoise	3.59E-14	−0.85	normal	2.80E-06	−3.01	down	PREDICTED: vacuolar iron transporter homolog 1 [Brassica rapa]
Bra015718.gene	VIT2	turquoise	3.83E-16	−3.22	down	−	−	−	PREDICTED: vacuolar iron transporter homolog 2-like [Brassica rapa]
Bra024856.gene	VIT1	blue	4.02E-16	2.19	up	−	−	−	PREDICTED: vacuolar iron transporter 1-like [Brassica rapa]
Bra025879.gene	VIT1	turquoise	3.45E-30	−3.17	down	−	−	−	PREDICTED: vacuolar iron transporter homolog 1-like [Brassica rapa]
Bra004127.gene	YSL7	yellow	3.68E-09	−1.18	down	2.85E-79	2.37	up	PREDICTED: probable metal-nicotianamine transporter YSL7 [Brassica rapa]
Bra009756.gene	YSL2	turquoise	3.37E-127	−2.09	down	1.00E-14	−3.86	down	PREDICTED: metal-nicotianamine transporter YSL2 [Brassica rapa]
Bra013764.gene	YSL1	brown	1.01E-08	−2.06	down	1.72E-101	−2.79	down	PREDICTED: metal-nicotianamine transporter YSL1 isoform X1 [Brassica rapa]
Bra019246.gene	YSL1	yellow	−	−	−	2.34E-23	1.52	up	PREDICTED: metal-nicotianamine transporter YSL1-like [Brassica rapa]
Bra039095.gene	YSL6	yellow	9.32E-02	0.14	normal	2.86E-63	1.19	up	BnaA09g01870D [Brassica napus]
Bra003226.gene	FER3	brown	6.74E-52	−2.78	down	7.09E-208	−3.61	down	PREDICTED: ferritin-3, chloroplastic [Brassica rapa]
Bra005677.gene	FER1	brown	5.90E-260	−2.36	down	4.05E-273	−1.44	down	PREDICTED: ferritin-1, chloroplastic [Brassica rapa]
Bra007215.gene	FER3	brown	9.58E-34	−1.36	down	0.00	−2.81	down	PREDICTED: ferritin-3, chloroplastic [Brassica rapa]
Bra029891.gene	FER2	blue	2.22E-26	4.19	up	−	−	−	PREDICTED: ferritin-2, chloroplastic [Brassica rapa]
Bra008385.gene	HA	green	2.85E-02	−0.10	normal	7.86E-135	1.05	up	PREDICTED: V-type proton ATPase catalytic subunit A-like [Brassica rapa]
Bra027752.gene	HA	turquoise	6.19E-77	−1.06	down	3.42E-56	−1.42	down	PREDICTED: V-type proton ATPase subunit E3 [Brassica oleracea var. oleracea]
Bra032402.gene	HA	blue	4.92E-39	5.24	up	−	−	−	PREDICTED: V-type proton ATPase subunit E2 [Brassica rapa]
Bra040512.gene	HA	turquoise	7.40E-33	−1.15	down	7.56E-02	−0.26	normal	PREDICTED: V-type proton ATPase subunit G1-like [Brassica rapa]
Bra000495.gene	FIT	blue	1.38E-59	3.26	up	−	−	−	PREDICTED: transcription factor FER-LIKE IRON DEFICIENCY-INDUCED TRANSCRIPTION FACTOR-like [Brassica rapa]
Bra011972.gene	FIT	blue	8.47E-98	2.75	up	−	−	−	PREDICTED: transcription factor FER-LIKE IRON DEFICIENCY-INDUCED TRANSCRIPTION FACTOR [Brassica rapa]
Bra034392.gene	FIT	blue	2.50E-30	1.00	up	−	−	−	PREDICTED: transcription factor FER-LIKE IRON DEFICIENCY-INDUCED TRANSCRIPTION FACTOR-like [Brassica rapa]
Bra015415.gene	ZIP5	black	5.26E-11	2.64	up	9.84E-09	2.34	up	PREDICTED: zinc transporter 5 isoform X1 [Brassica rapa]
Bra020314.gene	ZIP2	turquoise	4.13E-06	−1.12	down	−	−	−	PREDICTED: zinc transporter 2 [Brassica rapa]
Bra030826.gene	ZIP11	brown	6.10E-05	−1.28	down	5.22E-46	−2.04	down	PREDICTED: zinc transporter 11 [Brassica rapa]
Bra008437.gene	NRAMP1	red	9.81E-94	1.70	up	3.72E-39	1.24	up	PREDICTED: metal transporter Nramp1 [Brassica rapa]
Bra012154.gene	NRAMP4	cyan	0.00	2.26	up	1.12E-126	1.36	up	PREDICTED: metal transporter Nramp4 [Brassica rapa]
Bra040711.gene	NRAMP2	green	8.09E-02	−0.44	normal	4.49E-07	−1.06	down	PREDICTED: metal transporter Nramp2-like [Brassica rapa]
Bra040573.gene	OPT3	black	0.00	4.26	up	3.00E-303	1.43	up	PREDICTED: oligopeptide transporter 3 [Brassica rapa]
Bra005150.gene	Ctr4	turquoise	3.02E-06	−1.21	down	−	−	−	PREDICTED: copper transporter 4 [Brassica rapa]

*CK root vs. Fe root: the treatment of iron deficiency in roots was compared with the normal treatment; CK leaf vs. Fe leaf: the treatment of iron deficiency in leaf was compared with the normal treatment; DESeq2_ FDR, false discovery rate of differential gene expression; up, up regulation; down, down regulation; normal, has no change.*

### Differentially Expressed Genes Related to Photosynthesis

Based on the functional annotation of 8148 DEGs, 38 DEGs implicated in photosynthesis were searched, as yellowing of seedlings under iron deficiency stress could impair photosynthesis. These genes include 10 chlorophyll a/b binding protein, 3 carotenoids, 3 glutamine-tRNA reductase, 2 vetiver diphosphate reductase, and 19 light system I and II subunit protein genes. We discovered 31 genes that are part of the MEbrown module among these 38 genes (Supplementary Table 6). Thus, we hypothesize that these 31 genes may play a critical role in controlling leaf color in seedlings under iron deficiency stress, based on the highly significant positive correlation between Chlab and MEbrown modules (Figure 6).

### The Interaction Network Analysis

**FIGURE 7:**
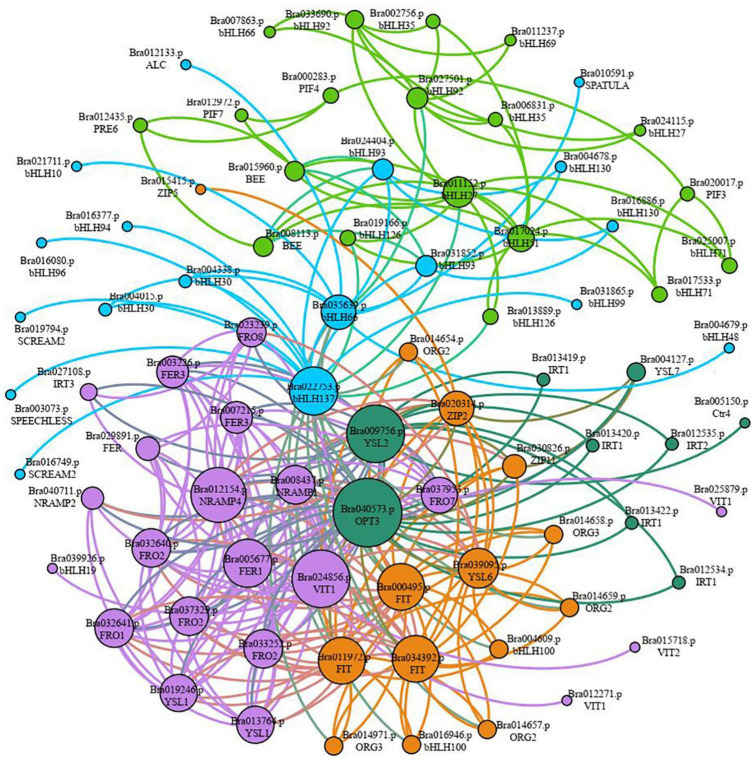
Classification of interaction networks between iron absorption and transport-related proteins and key transcription factor bHLH. Each interactive network system is represented by different colors (purple, dark green, orange, light blue, and green), and the size of circles represents the strength of the interaction. The larger the circle is, the stronger the interaction is. On the contrary, the weaker the interaction is.

Sixty-seven gene families were discovered to be involved in the control of iron deficiency stress by examining differentially expressed transcription factors, including the WRKY, MYB, AP2/ERF-ERF, bHLH, NAC, C2H2, bZIP, HB-HD-ZIP, and GARP-G2-like families (Supplementary Figure 2). The WRKY, MYB, ERF, bHLH, NAC, C2H2, and bZIP families account for 50.35% of the genes. Among them, 67 *bHLH* family genes were shown to be involved in iron deficiency stress (Supplementary Figure 2 and Supplementary Table 8). Given the importance of *bHLH* family genes in iron stress, we utilized STRING to examine the interaction network of 67 bHLH transcription factors, 40 iron absorption and transport-related proteins in Table 2, and the link between the two categories of proteins. According to the results of the protein interaction network prediction, these proteins were split into five categories, each of which was represented by a distinct color (Figure 7). One category was formed by seventeen iron absorption and transport proteins and one bHLH transcription factor (Bra039926) (purple). Four iron absorption and transport proteins, as well as 10 bHLH genes, were grouped (orange). Nine iron absorption and transport proteins were grouped in one category (dark green), whereas the remaining bHLH family genes were split into two groups (light blue and green). Three *FIT* genes (*Bra000495*, *Bra011972*, and *Bra034392*) belonging to the bHLH family transcription factors, which are iron deficiency-induced transcription factors, were discovered in the orange interaction network diagram. Three *FIT* genes had 83.75 to 86.88% sequence similarity with *Arabidopsis FIT* (*bHLH029*). Bra014657, Bra014658, Bra014659, Bra014971, Bra016946, and Bra004609 are the three FIT proteins that interact with Bra014657, Bra014658, Bra014659, Bra014971, Bra016946, and Bra004609 (Supplementary Figure 3). Since *Arabidopsis FIT* (*bHLH029*) interacts with *bHLH038* and *bHLH100* to form heterodimers. Similarly, *Arabidopsis FIT* (*bHLH029*) starts the expression of *FRO2* and *IRT1* genes in roots to promote iron absorption (Colangelo and Guerinot, 2004; Wang et al., 2013). While *Bra014657*, *Bra014658*, *Bra014659*, and *Bra014971* are homologous genes of *Arabidopsis bHLH38* and *Bra016946* and *Bra004609* are homologous genes of *bHLH100*. As a result, we hypothesized that the three FIT proteins, in conjunction with other bHLH transcription factors, would help maintain the iron balance in non-heading Chinese cabbage.

### RNA-Seq Verification Based on Quantitative Real-time PCR

To ensure the accuracy of the transcriptome data, we used qRT-PCR to confirm the 9 candidate genes. The results revealed that these 9 genes were consistently up-regulated and down-regulated in roots and leaves, which matched the transcriptome sequencing findings (Supplementary Table 9). The Pearson correlation coefficient (*R*^2^) was used to analyze the correlation between transcriptome data and qRT-PCR data, and the results showed that the *R*^2^ was 0.8025, indicating that the RNA sequencing data and qRT-PCR data were positively correlated (Supplementary Figure 4). We conclude that these differentially expressed genes may play an important part in iron deficiency stress based on our findings.

## Discussion

Due to the obvious large loss in chlorophyll as a result of iron deficiency, plants growing in alkaline and calcareous soils face nutritional problems (Schenkeveld et al., 2008). Crop yields and varieties suffer significant economic losses due to iron deficiency (Schenkeveld et al., 2008). Iron deficiency is commonly associated with conditions such as high pH and high bicarbonate concentrations (Marschner et al., 2011; Kobayashi et al., 2014). As a result, non-heading Chinese cabbage grown in alkaline soil in the north is susceptible to iron deficiency browning of young leaves (Figure 1). Dicots induce the roots to release protons that acidify the surrounding soil to better adapt to iron deficiency stress (Marschner et al., 2011). Roots not only exude protons, but also organic compounds like malic acid and citric acid during the cation absorption process (Kobayashi et al., 2014). To better adapt to iron deficiency stress, Fe^3+^ was reduced to Fe^2+^ by iron chelate reductase found in the root plasma membrane (Robinson et al., 1999). There are numerous adaptations in non-heading Chinese cabbage to better adapt to iron deficiency stress.

### Removal of Reactive Oxygen Species by Antioxidant Enzyme System in Non-heading Chinese Cabbage Under Iron Deficiency Stress

When plants are exposed to abiotic stress, reactive oxygen species (ROS) build up in the cells, causing catastrophic damage. ROX in plants, for example, damage the photosynthetic organs of apple leaves when there is an iron deficiency (Cheng et al., 2020). Antioxidant enzyme systems (such as SOD, POD, and CAT) were discovered to be able to scavenge ROS (Liu et al., 2016; Aung and Masuda, 2020; Fan et al., 2021). Under iron deficiency stress, DEGs for POD, SOD, and CAT were also identified in the roots and leaves of non-heading Chinese cabbage (Table 1). There were 58 POD DEGs in the roots of non-heading Chinese cabbage under iron shortage stress, with 47 *POD* genes considerably repressed and 11 genes significantly upregulated. Iron deficiency stress generated three *SOD* genes and two *CAT* genes in the leaves (Table 1). These genes were found to be involved in peroxisome biosynthesis (ko00940) and phenylpropanoid biosynthesis (ko00940) in the KEGG pathway (ko04146). Hence, this is assumed that these antioxidant enzymes or secondary metabolites interact with ROS in many tissues, improving iron deficiency stress tolerance.

### Effect of Iron Deficiency on Plant Hormone Signal Transduction in Non-heading Chinese Cabbage

According to research findings, when individuals are iron deficient, the amount of IAA in their sunflower roots increases dramatically (Marschner et al., 1986). Exogenous IAA increased ethylene and NO levels in plants, whereas the ethylene signal transcription factors EIN3 (ETHYLENE IN-SENSITIVE3) and EIL1 (EIN3-LIKE1) worked together with FIT to positively regulate *AtFIT1* transcription, boosting iron absorption (Brumbarova et al., 2014). *EIN3-like3* (*Bra003831*) expression was also shown to be highly up-regulated in leaves in this investigation (Supplementary Table 5). Furthermore, we discovered that numerous auxin-related genes were implicated in the response in roots and leaves under iron shortage stress (Supplementary Table 5). As a result, we hypothesized that IAA may play a signal transduction role in the iron deficiency stress of non-heading Chinese cabbage leaves. Furthermore, we discovered that under iron deficiency stress, the expression of one *JAR* gene (*Bra034205*) related to jasmonic acid signal was significantly down-regulated in the roots and leaves of non-heading Chinese cabbage, which is similar to JA as a negative regulator involved in regulating iron uptake in *Arabidopsis* (Brumbarova et al., 2014; Kobayashi et al., 2016; Cui et al., 2018). We deduce from the above research that the iron nutrition regulatory signal in non-heading Chinese cabbage under iron deficiency stress is highly complex and that a range of hormone signaling molecules is involved.

### Pathways Analysis of Iron Absorption and Transport-Related Genes in Roots and Leaves of Non-heading Chinese Cabbage Under Iron Deficiency Stress

**FIGURE 8:**
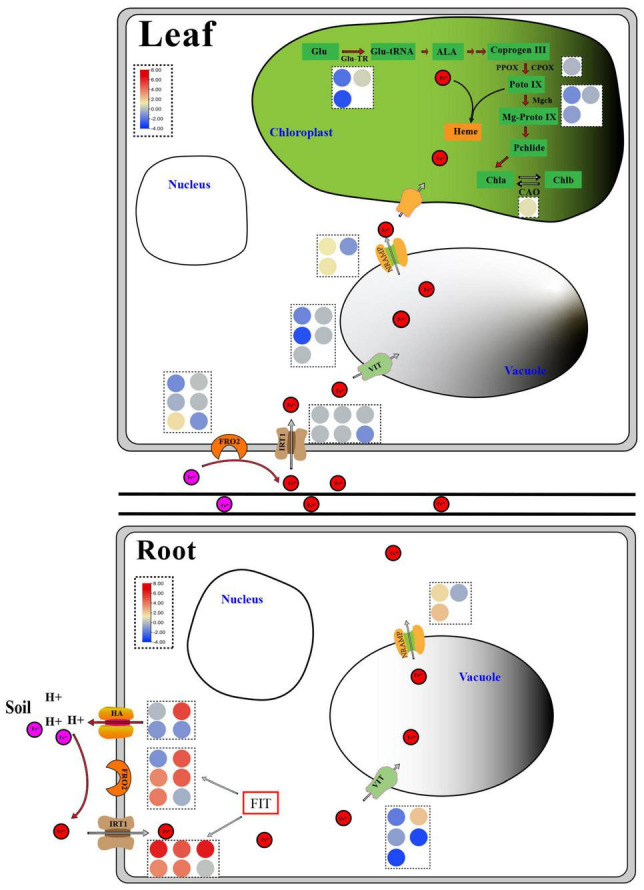
Response diagram of key genes in roots and leaves of non-heading Chinese cabbage under iron deficiency stress. The red circle represents Fe^2+^, the pink circle represents Fe^3+^, and the circles of different colors in the dotted box represent the FPKM values of key genes related to iron absorption and transport and chlorophyll metabolism, which are expressed in the form of heat map. Glu, Glutamic acid; Glu-TR, Glutamyl-tRNA reductase; Glu-tRNA, Glutamyl-tRNA; ALA, Aminolevulinic acid; Coprogen III, Coproporphyrinogen III; PPOX, Protoporphyrinogen oxidase; CPOX, Coproporphyrinogen III oxidase; Proto IX, Protoporphyrinogen IX; Mgch, Magnesium chelatase; Mg-Proto IX, Mg-Protoporphyrin IX; Pchlide, Protochlorophyllide; Chla, Chlorophyllide a; Chlb, Chlorophyllide b; CAO, Chlorophyllide a oxygenase; HA, H^+^-ATPase; FRO2, Ferric reduction oxidase 2; IRT1, Iron-regulated transporter 1; VIT, Vacuolar iron transporter; NRAMP, natural resistance-associated macrophage protein; FIT, Fer-like Fe deficiency-induced transcription factor.

The control of iron homeostasis in plants necessitates the involvement of several iron absorption and transport-related proteins and regulatory factors (Li et al., 2021). Ferric reduction oxidase (FRO2), iron transporter gene (IRT1), and H^+^-ATPase (HA) protein are among the proteins involved in iron uptake and transport on the cell membrane, as shown in Figure 8. *FRO2* and *IRT1* were the first *Arabidopsis thaliana* genes to be cloned in the 1990s. Under iron stress, they are engaged in iron intake and transport (Eide et al., 1996; Robinson et al., 1999). Under iron deficient conditions, cucumber and *Arabidopsis* induced a significant number of *HA* gene expressions, which played an important role in response (Santi et al., 2005; Santi and Schmidt, 2009). The expression levels of four *FRO*, five *IRT*, and one *HA* genes in the roots of non-heading Chinese cabbage were likewise found to be considerably up-regulated under iron deficiency stress in this study. Under iron deficiency stress, however, the expression levels of these genes in the leaves of non-heading Chinese cabbage were not significantly elevated or inhibited (Figure 8). Given that plants move the iron from underground to aboveground portions, we predicted that under iron deficiency, the leaves of non-heading Chinese cabbage were inhibited through the *FRO2*, *IRT1*, and *HA* genes, reducing iron absorption.

*AtVIT1* was discovered to be involved in the transport of iron ions to vacuoles in *Arabidopsis* (Kim et al., 2006), whereas *AtNRAMP3* was found to be involved in the transport of iron ions from vacuoles to cytoplasm and was activated under iron deficient stress (Lanquar et al., 2005). Under iron deficiency stress, 5 *VITs* and 3 *NRAMPs* were discovered in this study (Figure 8 and Table 2). Thus, we suggested that the plant would use *VIT* and *NRAMP* on the vacuole membrane in roots and leaves to cooperatively regulate the steady state of iron ions.

Ferritin (FER) is a polymeric iron storage protein that can store up to 4500 iron atoms in its central cavity and is essential for cell iron homeostasis (Zielińska-Dawidziak, 2015). In the early stages of photosynthesis, ferritin in leaves serves as an iron source for the synthesis of iron-containing proteins (Briat and Lobréaux, 1997). In *Arabidopsis*, four ferritin genes (*AtFER1-4*) were discovered for the first time. Only *AtFER2* was found in seeds, while *AtFER1*, *AtFER3*, and *AtFER4* were found in vegetative and reproductive tissues (Petit et al., 2001). Ferritin deficiency in *Arabidopsis* leaves reduces leaf development and CO_2_ fixation (Ravet et al., 2009). Under iron deficiency stress, the expression of three *FER* genes in roots and leaves was dramatically down-regulated, implying that iron deficit had a considerable impact on plant iron storage. To manage internal iron homeostasis, multi-gene co-expression was necessary to store and distribute iron ions between vacuoles and cytoplasm.

*FIT* (FER-LIKE FE DEFICIENCY-INDUCED TRANSCRIPTION FACTOR) is a key player in keeping iron levels in plant roots stable and limited (Colangelo and Guerinot, 2004; Yuan et al., 2005). *FIT* activity is produced not only by *ZAT12*, *DELLA*, and jasmonic acid but also by the interaction between bHLH and BTSL2 proteins, according to research (Birte and Petra, 2020). In *Arabidopsis thaliana*, *FIT* (*bHLH029*) formed heterodimers with the bHLH Ib subfamily members *bHLH038*, *bHLH039*, *bHLH100*, and *bHLH101*, and subsequently induced the expression of *FRO2* and *IRT1* genes in roots to enhance iron absorption (Colangelo and Guerinot, 2004; Wang et al., 2013). The level of Fe^2+^ in stems rose after overexpression of *bHLH039* in *Arabidopsis thaliana*, which was linked to the inhibition of *FRO3* and *NRAMP4* and the significant induction of *IRT1*, *FRO2*, *At3g07720*, and *IRT2* (Naranjo-Arcos et al., 2017; Birte and Petra, 2020). This finding implies that the bHLH Ib protein responds in a cascade to Fe-deficiency signals and that iron acquisition and dynamic balance in plants are regulated by a complex network. In this study, we discovered three *FIT* genes in the roots and leaves of non-heading Chinese cabbage under iron deficiency stress. These three FIT proteins interacted with bHLH38 analogous proteins (Bra014657, Bra014658, Bra014659, and Bra014971) and bHLH100 homologous proteins (Bra016946 and Bra004609) in *Arabidopsis*, according to the protein interaction network (Figure 7 and Supplementary Figure 3). OsbHLH064 and bHLH IVc transcription factors (OsbHLH057/PRI4, OsbHLH058/PRI2, OsbHLH059/PRI3, and OsbHLH060/PRI1) are also regulatory factors for iron absorption in rice (OsbHLH057/PRI4, OsbHLH058/PRI2, OsbHLH059/PRI3, and OsbHLH060/PRI1) (Kobayashi et al., 2019; Zhang et al., 2020). Iron absorption in tomatoes is controlled by Ib *SlbHLH068* and *FER* (Du et al., 2015). Furthermore, under iron deficiency stress, the transcription levels of these six bHLH transcription factors in roots and leaves were dramatically up-regulated. Given the discovery of 67 bHLH transcription factors, we predicted that bHLH transcription factors played a key regulatory role in t iron deficiency stress.

Plants with an iron deficit turn green due to a drop in chlorophyll levels (Donnini et al., 2009). Although the chlorophyll a-oxygenase (*CAO*) gene was dramatically upregulated in the chlorophyll metabolic pathway under iron deficiency stress, the glutamine-tRNA reductase (*Glu-TR*), co-proporphyrinogen oxidase (*CPOX*) gene, and Mg-chelating enzyme H subunit (*MgCh*) gene were all strongly suppressed (Figure 8). As a result, we hypothesized that the combined effect of these genes resulted in a considerable drop in chlorophyll a/chlorophyll b in non-heading Chinese cabbage leaves under iron deficiency stress. Photosynthetic activity and leaf chlorophyll were both reduced. The reduction of the light capture complex (LHC) is linked to this phenomenon (Donnini et al., 2009). Light capture of chlorophyll a/b binding protein (antenna protein) has been shown to improve light absorption and excitation energy transfer in studies (Li et al., 2008). Under iron deficiency stress, the expression of four chlorophyll a/b binding protein genes was dramatically down-regulated in non-heading Chinese cabbage leaves. In addition, 19 photosynthesis-related genes, including subunit I and subunit II, were shown to be considerably down-regulated in leaves (Supplementary Table 7).

## Conclusion

As shown in Figure 8, under iron deficiency stress, non-heading Chinese cabbage secretes H^+^ to the rhizosphere, acidifies the rhizosphere soil, increases the solubility of Fe^3+^ in the soil, lowers Fe^3+^ to Fe^2+^ through *FRO*, and enters the cells through iron transporters *IRT1*, *FRO2*, and *HA* on the cell membrane. Other iron transporter genes, hormone signaling control genes, active oxygen scavenging enzyme genes, chlorophyll synthesis pathway-related enzymes, and different transcription factors all contribute to Fe^2+^ entering the body and maintaining iron homeostasis in the body. Hence, these findings provide a theoretical foundation for future research into the molecular mechanisms of iron absorption and transport essential genes, and they are crucial for improving iron-deficient varieties genetically.

## Data Availability Statement

The original contributions presented in the study are included in the article/Supplementary Material. Transcriptome data have been submitted to the National Center for Biotechnology Information (NCBI) Sequence Read Archive (SRA) (Registration number PRJNA745363, https://www.ncbi.nlm.nih.gov/bioproject?term=PRJNA745363).

## Author Contributions

CS and JY contributed to the experiment design. DL, FP, NK, and HY performed the material preparation and data collection. WG, BC, XinzL, and JY analyzed the data. DL, CS, XinL, and CW wrote the first draft of the manuscript. All authors commented on previous versions of the manuscript, and read and approved the final manuscript.

## Conflict of Interest

The authors declare that the research was conducted in the absence of any commercial or financial relationships that could be construed as a potential conflict of interest.

## Publisher’s Note

All claims expressed in this article are solely those of the authors and do not necessarily represent those of their affiliated organizations, or those of the publisher, the editors and the reviewers. Any product that may be evaluated in this article, or claim that may be made by its manufacturer, is not guaranteed or endorsed by the publisher.

## Footnotes

1
https://www.ncbi.nlm.nih.gov/bioproject?term=PRJNA745363

2
https://urgi.versailles.inra.fr/download/iwgsc/IWGSC_RefSeq_Assemblies/v1.0/

## Supplementary Material

Supplementary Figure 1 Venn diagram of differentially expressed genes related to antioxidant enzymes in roots and leaves of non-heading Chinese cabbage. **(A)** Venn diagram of POD gene in roots and leaves of non-heading Chinese cabbage. **(B)** Venn diagram of SOD gene in roots and leaves of non-heading Chinese cabbage. **(C)** Venn diagram of CAT gene in roots and leaves of non-heading Chinese cabbage. **(D)** Venn diagram of APX gene in roots and leaves of non-heading Chinese cabbage.

Supplementary Figure 2 Statistics of the number of major transcription factors in differentially expressed genes.

Supplementary Figure 3 In bHLH transcription factors, the interaction network diagram of three *FIT* genes (*Bra000495*, *Bra011972*, and *Bra034392*) with other important proteins.

Supplementary Figure 4 Correlation analysis of FPKM values from transcriptome sequencing and the qRT-PCR values.
