# Supplementary figures and images for: Transcriptome Analysis Revealed the Molecular Response Mechanism of Non-heading Chinese Cabbage to Iron Deficiency Stress
Source: Front Plant Sci. 2022 Mar 11;13:848424. doi: 10.3389/fpls.2022.848424 (PMC8964371; doi:10.3389/fpls.2022.848424)

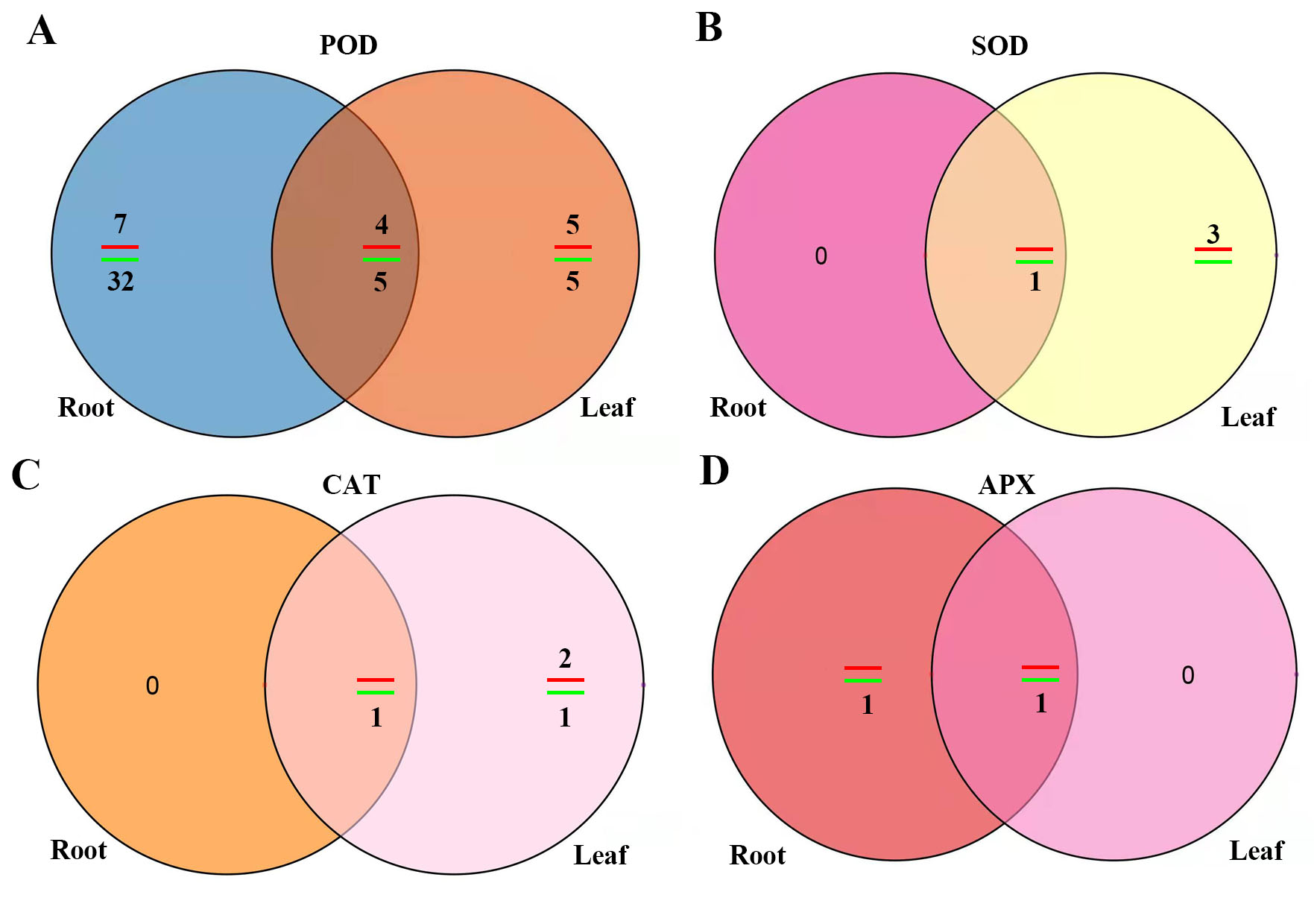

Supplement: Supplementary Figure 1 — Venn diagram of differentially expressed genes related to antioxidant enzymes in roots and leaves of non-heading Chinese cabbage. (A) Venn diagram of POD gene in roots and leaves of non-heading Chinese cabbage. (B) Venn diagram of SOD gene in roots and leaves of non-heading Chinese cabbage. (C) Venn diagram of CAT gene in roots and leaves of non-heading Chinese cabbage. (D) Venn diagram of APX gene in roots and leaves of non-heading Chinese cabbage. [file Image_1.JPEG]

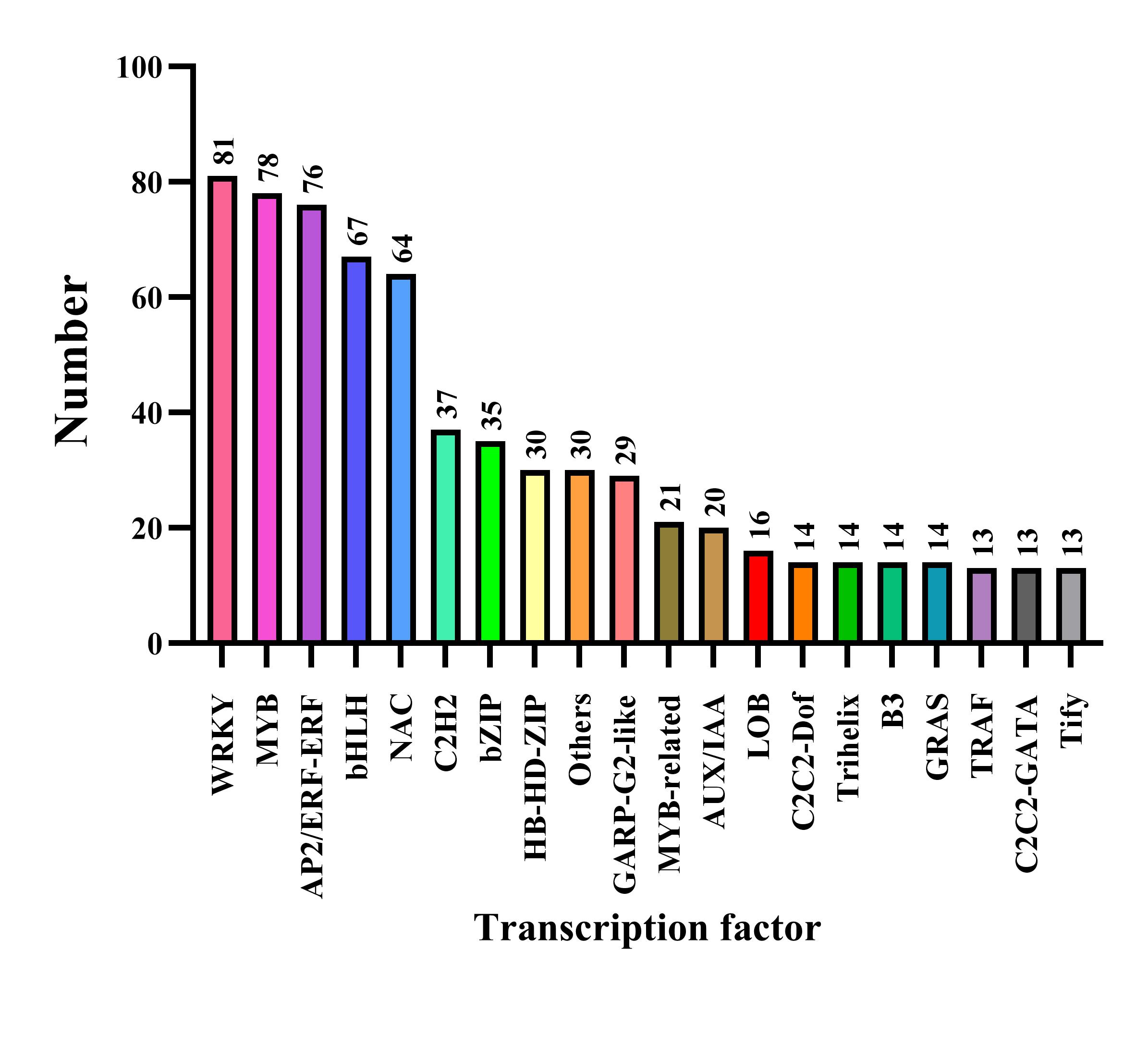

Supplement: Supplementary Figure 2 — Statistics of the number of major transcription factors in differentially expressed genes. [file Image_2.JPEG]

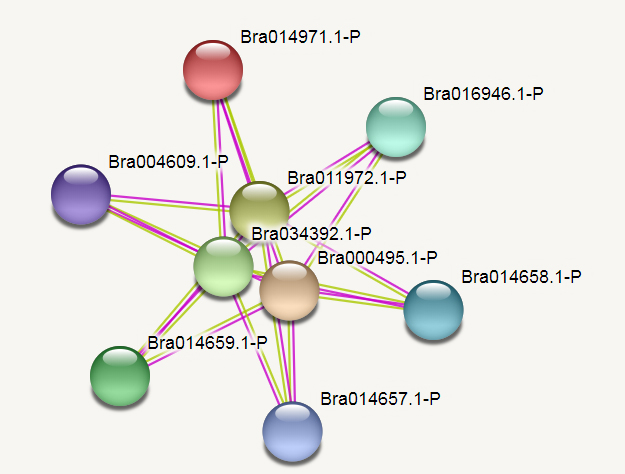

Supplement: Supplementary Figure 3 — In bHLH transcription factors, the interaction network diagram of three FIT genes (Bra000495, Bra011972, and Bra034392) with other important proteins. [file Image_3.JPEG]

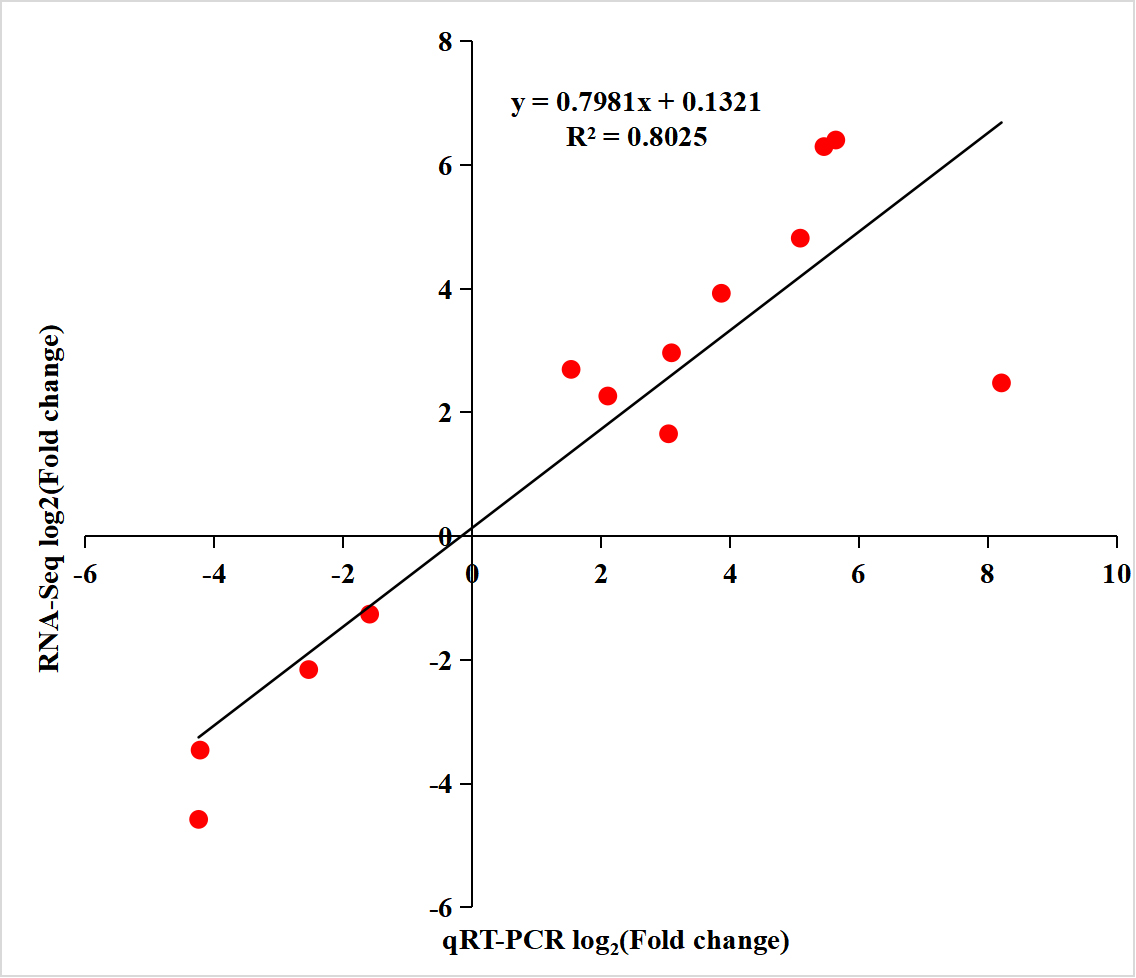

Supplement: Supplementary Figure 4 — Correlation analysis of FPKM values from transcriptome sequencing and the qRT-PCR values. [file Image_4.JPEG]
